# Multiple H^+^ sensors mediate the extracellular acidification-induced [Ca^2+^]_i_ elevation in cultured rat ventricular cardiomyocytes

**DOI:** 10.1038/srep44951

**Published:** 2017-03-23

**Authors:** Yuan-Lang Hu, Xue Mi, Chao Huang, Hui-Fang Wang, Jian-Ren Song, Qing Shu, Lan Ni, Jian-Guo Chen, Fang Wang, Zhuang-Li Hu

**Affiliations:** 1 Department of Pharmacology, School of Basic Medicine, Tongji Medical College, Huazhong University of Science and Technology, Wuhan, Hubei 430030, China; 2 The Key Laboratory of Neurological Diseases (HUST), Ministry of Education of China, Wuhan, Hubei 430030, China; 3 The Key Laboratory for Drug Target Research and Pharmacodynamic Evaluation of Hubei Province, Wuhan 430030, China

## Abstract

Acidosis has been known to cause “Ca^2+^ transients”, however, the mechanism is still uncertain. Here, we demonstrated that multiple H^+^ sensors, such as ASICs, TRPV1 and proton-sensing G protein coupled receptors (GPCRs) are involved in extracellular acidification-induced intracellular calcium ([Ca^2+^]_i_) elevation. By using calcium imaging measures, we observed that both ASIC and TRPV1 channels inhibitors suppressed the [Ca^2+^]_i_ elevation induced by extracellular acidosis in cultured rat cardiac myocytes. Then, both channels mRNA and proteins were identified by RT-PCR, western blotting and immunofluorescence. ASIC-like and TRPV1-like currents were induced by extracellular acidification, suggesting that functional ASIC and TRPV1 channels jointly mediated extracellular calcium entry. Furthermore, either pre-exhaustion of sarcoplasmic reticulum (SR) Ca^2+^ with thapsigargin or IP_3_ receptor blocker 2-APB or PLC inhibitor U73122 significantly attenuated the elevation of [Ca^2+^]_i_, indicating that the intracellular Ca^2+^ stores and the PLC-IP_3_ signaling also contributed to the acidosis-induced elevation of [Ca^2+^]_i_. By using genetic and pharmacological approaches, we identified that ovarian cancer G protein-coupled receptor 1 (OGR1) might be another main component in acidosis-induced release of [Ca^2+^]_i_. These results suggest that multiple H^+^-sensitive receptors are involved in “Ca^2+^ transients” induced by acidosis in the heart.

Acidosis is a noxious stimulus that often comes from inflammation, ischemia or other pathological conditions. It is known to cause “Ca^2+^ transients” and lead to tissue injury^1^,^2^. However, the concrete mechanisms of Ca^2+^ transients are diverse and uncertain. Several studies have shown that the increase of [Ca^2+^]_i_ is induced by the influx of extracellular Ca^2+^ via membrane Ca^2+^ channels or Na^+^/Ca^2+^exchangers^3^,^4^. However, the increase of intracellular Ca^2+^ also can be resulted from the mobilization of sarcoplasmic reticulum (SR) during acidosis^5^,^6^. Besides above manners, another possible mechanism should not to be ignored, that is, the participation of H^+^-sensitive ion channels or receptors, such as acid-sensing ion channels (ASICs), transient receptor potentialvanilloid-1 (TRPV1) and a group of proton sensing G protein coupled receptors (GPCRs). All of them could be activated by acidosis and mediate “Ca^2+^ transients”.

ASICs belong to amiloride-sensitive epithelial sodium channel family in vertebrates and the degenerin family of *C. elegans*. Until now, the proton-sensitive members have found to be encoded by four different genes (ASIC1-ASIC4) with six protein subunits cloned, which are ASIC1a (ASIC or BNaC2), ASIC1b (ASIC1β), ASIC2a (MDEG, BNaC1), ASIC2b (MDEG2), ASIC3 (DRASIC) and ASIC4 (SPASIC)^7^,^8^,^9^. ASIC channels are activated by an extracellular drop of pH with a predominant permeability to Na^+^ ions. Some of ASIC subunits, such as homomeric ASIC1a^10^ and a third human ACCN2 transcript variant (hVariant 3)^11^ also carry Ca^2+^.

TRPV1 is a polymorphic sensor to sense various stimuli, such as pain, temperature, tension and acid^12^,^13^. It is a tetrameric membrane protein composing of four identical subunits, and each subunit contains six transmembrane regions to form a non-selective cation channel with high permeability to Ca^2+ ^14. Although TRPV1 is firstly identified in sensory neurons in 1997^15^, it is also identified in other tissues, especially in the cardiovascular system, including vascular smooth muscle cells, vascular endothelial cells^16^, and the mitochondria of cardiomyocytes^17^. Activation of TRPV1 channel by capsaicin has been reported to improve endothelium - dependent vasorelaxation and prevent hypertension^18^, Myocardial reperfusion injury is also reported to be mitigated by limiting the interaction between TRPV1 and calcineurin^17^.

Ovarian cancer G protein-coupled receptor 1 (OGR1), T cell death-associated gene 8 (TDAG8), G protein coupled receptor 4 (GPR4) and G2 accumulation (G2A) are all belong to proton-sensing GPCRs family, they share 40–50% homology with different action modes and signaling pathways^19^. Among them, OGR1 has been identified to be a novel drug target for ischemic heart disease^20^. As we know, protons could be accumulated under ischemia conditions because of tissue acidosis, thus, protons can function as signaling molecules to activate proton-sensing OGR1 and other similar GPCRs family in acidic extracellular pH.

Here, we investigate the relationship between cardiac “Ca^2+^ transients” and above H^+^-sensitive ion channels or receptors. During the process of diseases that cause acidification, ASICs, TRPV1 and proton-sensing GPCRs would sense external changes of pH value, then induce the opening of ion channels and influx of calcium ion, resulting in the activation of various intracellular signaling cascades. Therefore, cardiac H^+^ sensors will have an important significance in cardiac diseases.

## Results

### ASICs and TRPV1 jointly mediate extracellular acidification inducing calcium entry in cultured rat cardiac myocytes

**Figure 1:**
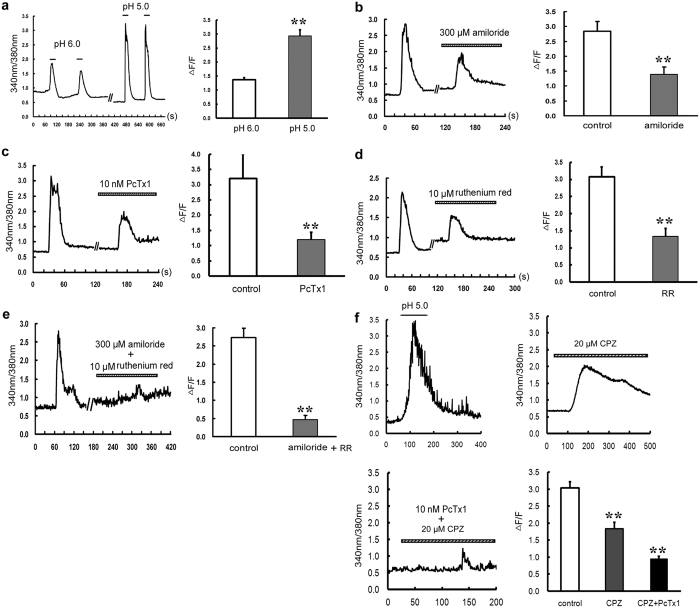
Inhibitory effect of ASIC and TRPV1 inhibitors on extracellular acid solution-induced [Ca^2+^]_i_ elevation in cultured rat ventricular cardiomyocytes. (**a**) Representative [Ca^2+^]_i_ responses and quantitative analysis of normalized Fura-2/AM fluorescence induced by pH 6.0 (n = 15 cells) and pH 5.0 (n = 19 cells) solutions. Data were expressed as mean ± s.e.m (**P < 0.01 *vs* pH 6.0, Student’s *t*-test). Pretreatment with (**b**) Amiloride (300 μM, n = 12 cells), (**c**) PcTx1 (10 nM, n = 12 cells, left), (**d**) Ruthenium red (10 μM, n = 16 cells) or (**e**) Amiloride + ruthenium red (n = 10 cells) for 5 min all inhibited [Ca^2+^]_i_ elevation. Data were shown as mean ± s.e.m. **P < 0.01 vs control (Student’s *t*-test). RR, ruthenium red. (**f**) Representative [Ca^2+^]_i_ responses and quantitative analysis of normalized Fura-2/AM fluorescence induced by pH 5.0 solutions (n = 20 cells, control), pretreatment with CPZ (10 μM, n = 17 cells) and CPZ + PcTx1 (10 nM, n = 23 cells) for 5 min. Data were shown as mean ± s.e.m. **P < 0.01 vs control (ANOAN followed by LSD).

Using ratio metric fura-2/AM recordings, we performed Ca^2+^ imaging measurements in cultured rat cardiac myocytes. As shown in Fig. 1a, a rapid and transient elevation of intracellular calcium ([Ca^2+^]_i_) was observed when the pH value of extracellular solution was rapidly lowered from 7.4 to 6.0 or 5.0. This elevation is reversible and could be stably induced when another acidosis solution was given again. The peak of ΔF/F was 1.37 ± 0.08 at pH 6.0 (n = 15 cells) and 2.93 ± 0.22 at pH 5.0 (n = 19 cells, *P* < 0.01 *vs* pH 6.0, Student’s *t*-test, Fig. 1a). These results suggest that extracellular acidification (pH 6.0 or pH 5.0) can elevate the [Ca^2+^]_i_ in primary cultured cardiomyocytes with a pH-dependent manner. We then chose pH 5.0 in the following experiments.

In order to explore the possibility of the involvement of ASICs and TRPV1 in the influx of extracellular Ca^2+^, we used pharmacological inhibitors for ASICs and TRPV1. It was shown that, bath application of ASIC blockers amiloride (300 μM, a nonspecific inhibitor of ASICs) inhibited the increase of [Ca^2+^]_i_ by 50.07 ± 5.70% (n = 12 cells, *P* < 0.01 *vs* control, Student’s *t*-test, Fig. 1b), and PcTx1 (10 nM, a specific inhibitor of ASIC1) inhibited the increase by 53.51 ± 3.31% (n = 12 cells, *P* < 0.01 *vs* control, Student’s *t*-test, Fig. 1c), while incubation of TRPV1 non-specific antagonist ruthenium red (10 μM) inhibited [Ca^2+^]_i_ rise by 56.16 ± 6.22% (n = 16 cells, *P* < 0.01 *vs* control, Student’s *t*-test, Fig. 1d). Then, we added amiloride (300 μM) and ruthenium red (10 μM) simultaneously and found that the Ca^2+^ elevation induced by pH drop was largely blocked, which was decreased by 82.61 ± 4.18% (n = 10 cells, *P* < 0.01 *vs* control, Student’s *t*-test, Fig. 1e). Since ruthenium red is also an effective blocker of ryanodine receptors, we chose CPZ, a specific inhibitor of TRPV1 to evaluate its effect on [Ca^2+^]_i_ elevation again. The inhibition ratio was 39.33 ± 5.91% with CPZ single treatment and it raised to 68.38 ± 2.03% when combining with PcTx1 (n = 17~23 cells, *P* < 0.01 vs control, ANOAN followed by LSD, Fig. 1f). These results indicate that both ASIC channels and TRPV1 channel are the main sources of [Ca^2+^]_i_ elevation in cultured rat cardiac myocytes.

### Expressions of ASICs in cultured rat cardiac myocytes

**Figure 2:**
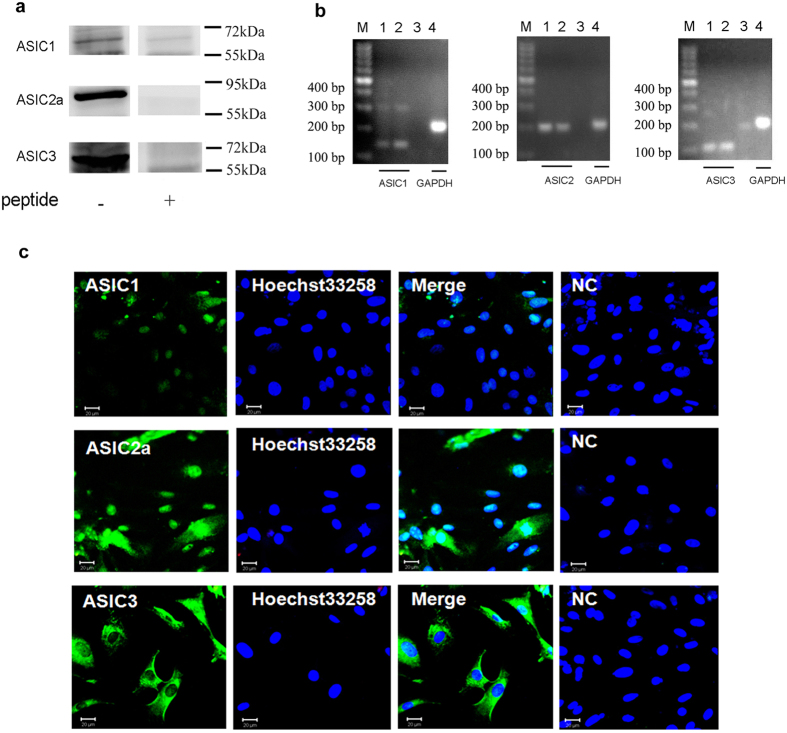
Expressions of ASIC subunits in rat cardiomyocytes. (**a**) Expressions of ASIC1, 2a and 3 proteins in rat cultured cardiomyocytes. Individual ASIC subunits peptides were added as negative controls. The blots were cropped from Supplementary Fig. S1a. The representative full-length blot for ASIC2a was shown in Supplementary Fig. S2. (**b**) Expressions of ASIC1, 2 and 3 transcripts in cultured rat ventricular myocytes. GAPDH transcript was used as control. M: marker; 1, 4: cardiomyocytes; 2: cortex; 3: negative control. (**c**) Double - labeling fluorescence of ASICs (*green*) and nucleus (*blue*, marker: Hoechst 33258) in cultured ratcardiomyocytes. NC: pretreatment with immunogenic peptide as negative control. Scale bars: 20 μm. All data were represented from at least three similar independent experiments.

In order to confirm whether ASIC channels are expressed on cardiac myocytes, specific antibodies were used for western blotting analysis. The results showed specific ASIC1, 2a and 3 bands in cultured rat cardiac myocytes, and the molecular weights of all ASIC subunits in rat heart cells were near 72 kDa. After adding peptides to react with respective antibodies, the corresponding bands almost disappeared (Fig. 2a and Supplementary Figs S1a and S2). We further performed RT-PCR to clarify ASICs gene expressions from cultured rat ventricular myocytes, the products corresponding to ASIC1, 2 and ASIC3 were similar between cardiomyocytes and brain cortex, which were 140, 203, and 107 bp, respectively (Fig. 2b). These results indicate that three ASIC subunits transcripts and proteins are expressed in rat cardiomyocytes.

Then, we observed the distribution of different ASIC proteins in the heart tissues by immunofluorescence. As shown in Fig. 2c, ASIC proteins were recognized by corresponding antibodies and the double immunofluorescence staining showed different spread immunosignals among three subunits in the cultured rat cardiomyocytes. ASIC1, but not ASIC3 protein predominantly merged with nuclear marker Hoechst 33258, ASIC2a had a uniform distribution, not only in nuclear but also in cytoplasm. The various distributions of ASICs subunits suggested the distinct intracellular functions and characteristics of ASICs on the heart.

### Characteristics of ASIC-like currents in cardiac myocytes

**Figure 3:**
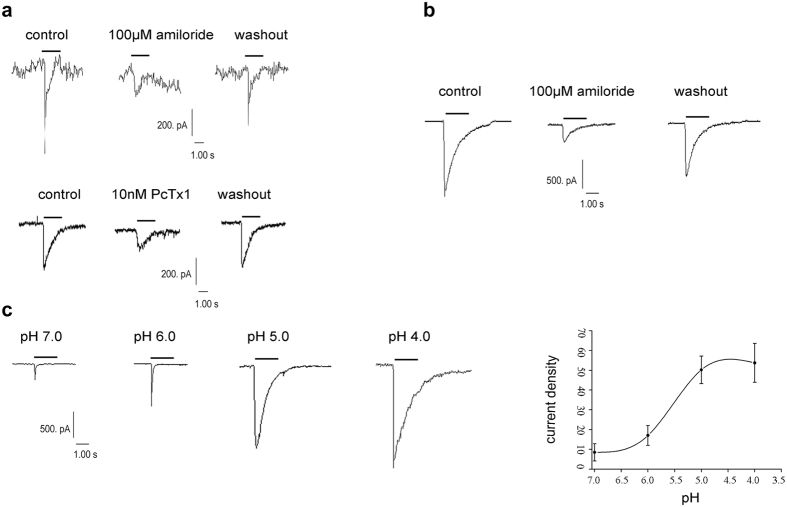
ASIC currents in cultured and acute isolated rat cardiomyocytes. (**a**) Representative ASIC-like currents inhibited by amiloride (100 μM, n = 3 cells) and PcTx1 (10 nM, n = 4 cells) in cultured rat cardiomyocytes. (**b**) Inhibition of ASIC-like currents by amiloride (100 μM, n = 3 cells) in acute isolated myocardiocytes of adult rat. (**c**) pH-dependent (upper) and pH-current density curve (lower) of ASIC currents in cultured rat cardiomyocytes. “—” indicates the duration of pH = 7.0, 6.0, 5.0 or 4.0 from 7.4. pH 7.0: 8.51 ± 4.37 pA/pF (n = 8 cells), pH 6.0: 17.03 ± 5.02 pA/pF (n = 11 cells), pH 5.0: 50.20 ± 6.94 pA/pF (n = 7 cells), pH 4.0: 53.73 ± 9.87 pA/pF (n = 7 cells). Each point represented as the mean ± s.e.m.

The recorded currents were induced by extracellular acidosis in cultured rat cardiomyocytes. At a holding potential of −80 mV, a rapid reduction of extracellular pH to 6.0 resulted in a fast activating ASIC-like inward current. These currents could be blocked by 100 μM amiloride and 10 nM PcTx1, and the amplitude decreased by 71.33 ± 3.01% (n = 3 cells, *P* < 0.05 *vs* control, Student’s *t*-test) and 46.67 ± 4.25% (n = 4 cells, *P* < 0.05 *vs* control, Student’s *t*-test), respectively. The inhibitory effect was mostly recovered after washout (Fig. 3a), further confirming that these currents were ASIC-like currents.

In order to eliminate the influence of culture conditions and developmental stage, we also recorded ASIC-like currents in adult rat acute isolated ventricular cardiocytes. As shown in Fig. 3b, the amplitude of inward current was inhibited by 74.21 ± 3.34% (n = 3 cells, *P* < 0.05 *vs* control, Student’s *t*-test) with 100 μM amiloride, and recovered mostly after washout. Thus, ASIC currents in cardiomyocytes displayed similar electrophysiological property as that in nervous system. Local decrease in extracellular solution to pH 7.0 was sufficient to induce an ASIC-like current, and the amplitudes of ASIC current were increased gradually along with the decrease in pH from 7.4 to 7.0, 6.0, 5.0 and 4.0. Fitting with Hill equations, the curve of pH-current density was “S” shape, and the pH_50_ (pH for half-maximal activation) was 5.73 ± 0.18 (n = 7~11 cells, Fig. 3c). These results indicate that ASIC currents in the rat cardiomyocytes are pH-dependent.

### Expression and characteristic of TRPV1 channel in rat cardiac myocytes

**Figure 4:**
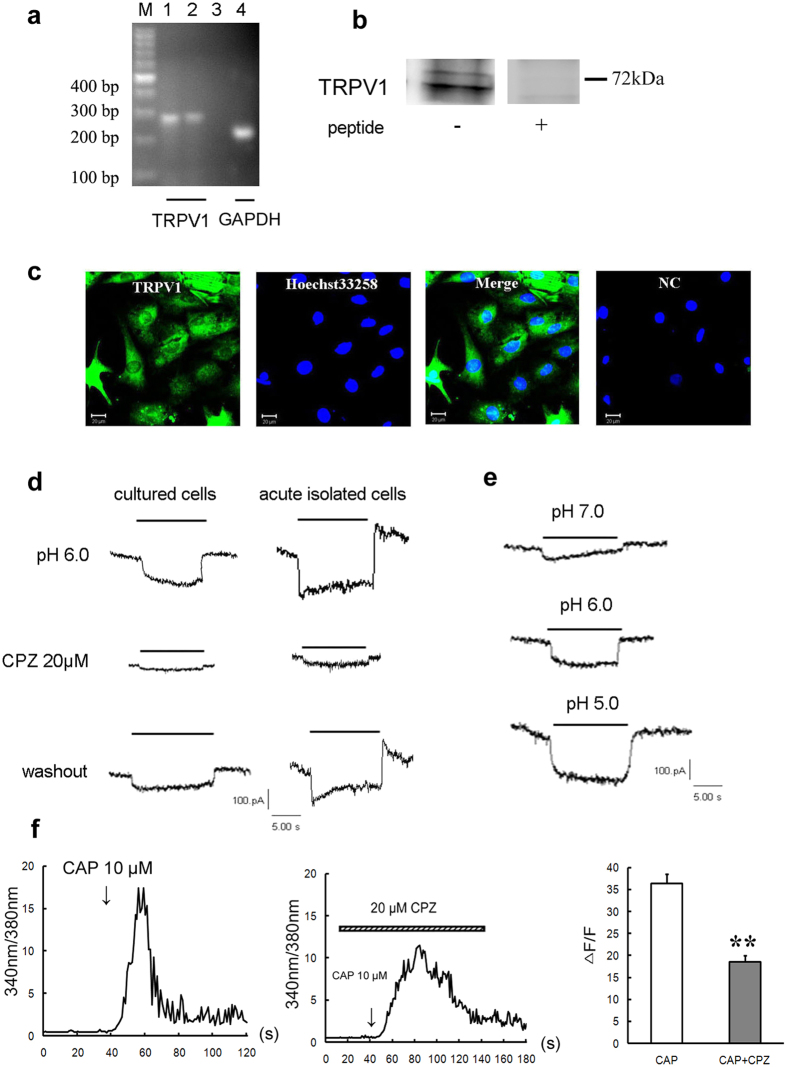
Functional expression of TRPV1 channel in rat cardiomyocytes. (**a**) RT-PCR detection of TRPV1 mRNA expressions in cultured rat ventricular cardiocytes. GAPDH were used as positive controls. M: marker; 1, 4: cardiomyocytes; 2: cortex; 3: negative control. (**b**) Western blotting indicating the protein expression of TRPV1 in cultured ventricular cardiocytes of rat. TRPV1 peptide was used as negative control. The blots were cropped from Supplementary Fig. S1b. (**c**) Double immunostaining of TRPV1 (*green*) and nucleus (*blue*, marker: Hoechst33258) in rat cultured cardiomyocytes. NC: pretreatment with immunogenic peptide as negative control. Scale bars: 20 μm. Above all data were represented from three similar independent experiments. (**d**) TRPV1-like currents reversibly inhibited by CPZ (20 μM, n = 3 cells) in cultured and acute isolated rat cardiomyocytes. (**e**) Representative TRPV1 current traces evoked by indicated pH solutions from pH 7.4 in cultured rat cardiomyocytes. “—” indicates the duration of pH = 7.0, 6.0 or 5.0. (**f**) Representative [Ca^2+^]_i_ responses and quantitative analysis of normalized Fura-2/AM fluorescence induced by capsaicin (10 μM, n = 16 cells) and CPZ (20 μM) + capsaicin (10 μM, n = 16 cells). Data were shown as mean ± s.e.m. **P < 0.01 vs capsaicin (Student’s *t*-test). CAP: capsaicin.

Meanwhile, another H^+^-sensitive channel, TRPV1 channel was detected. Firstly, TRPV1 transcripts were shown in cultured cardiocytes of rat as well as in cortex, the products were at 282 bp (Fig. 4a). Then at the protein level, the single prominent bands of 55~72 kDa were recognized in cultured ventricular myocytes. The detected TRPV1 protein should be specific because adding homologus peptide led to negative consequence (Fig. 4b and Supplementary Fig. S1b).Subsequently, the double immunostaining results showed that TRPV1 protein evenly expressed in rat ventricular myocytes. Using Hoechst 33258 as a nuclear indicator, we found that TRPV1 protein was distributed not only in the nucleus but also in the plasma of cardiac cells (Fig. 4c).

Then, we investigated the electrical properties of TRPV1 channel in cultured and acute isolated cardiomyocytes. TRPV1-like currents were recorded at a holding potential of −80 mV by a rapid reduction of extracelluar pH from 7.4 to 6.0, and they were activated rapidly and sustained without desensitization until extracellular pH returned to 7.4. The currents in both kinds of cells could be reversibly blocked by capsazepine (CPZ, 20 μM), a neuronal TRPV1-specific inhibitor (n = 3 cells, *P* < 0.05 *vs* control, Student’s *t*-test, Fig. 4d), indicating that the sustained currents are actually mediated by TRPV1. Along with the pH value decreasing from 7.4 to 7.0, 6.0, 5.0, the current amplitude was increased gradually (Fig. 4e). In order to demonstrate the presence of TRPV1, capsaicin, a specific agonist of TRPV1 channel was used here. A robust elevation of [Ca^2+^]_i_ was seen by capsaicin (10 μM, n = 16 cells) stimulation and CPZ (20 μM) blocked it largely (n = 16 cells, *P* < 0.01 *vs* control, Student’s *t*-test, Fig. 4f). Thus, other than ASICs, TRPV1 channel was another H^+^-sensitive channel in the heart.

To summarize the ratios, we found that after exposure to extracellular pH 6.0, 34.25% in cultured ventricular cells and 23.08% in acute-isolated ventricular cells displayed TRPV1 - like currents, while ASIC - like currents in these two kinds of cells took up to 43.83% and 61.54%, respectively, the remaining cells had no response to extracellular acidosis.

### Phospholipase C (PLC) -inositol 1,4,5-trisphosphate (IP_3_) signaling and sarcoplasmic reticulum (SR) mobilization mediate acidosis-induced Ca^2+^ elevation in the absence of extracellular Ca^2+^

**Figure 5:**
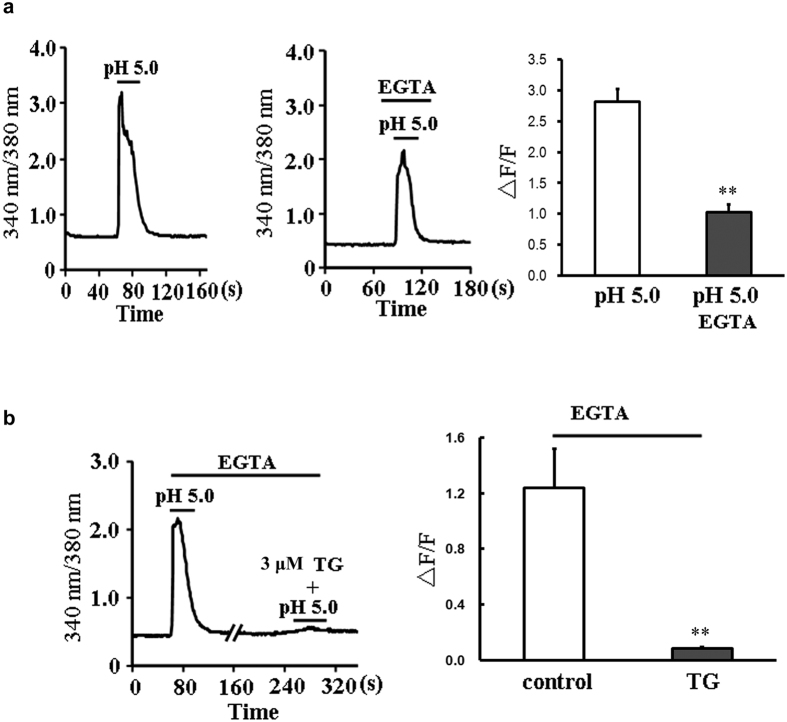
SR mobilization mediates the acidosis-induced [Ca^2+^]_i_ elevation when extracellular Ca^2+^ was removed from cardiomyocytes. (**a**) Representative traces of 340/380 nm ratio and summary data (∆F/F) of primary cultured cardiomyocytes showing pH 5.0 solution-induced [Ca^2+^]_i_ elevation in the presence (n = 25 cells) or absence (with EGTA, n = 19 cells) of extracellular Ca^2+^. Data were shown as mean ± s.e.m (**P < 0.01 *vs* pH 5.0 with extracellular Ca^2+^, Student’s *t*-test). (**b**) Representative 340/380 nm ratio and summary data (∆F/F) of primary cultured cardiomyocytes showing pH 5.0 solution-induced [Ca^2+^]_i_ elevation in the presence (n = 14 cells) or absence (n = 15 cells) of TG (3 μM). Data were shown as mean ± s.e.m (**P < 0.01 *vs* control, Student’s *t*-test).

Although ASIC channels and TRPV1 channel were the main sources of [Ca^2+^]_i_ elevation in cultured rat cardiac myocytes, both specific inhibitors could not block acidosis-induced Ca^2+^ elevation completely, indicating that other mechanism should be involved. Here, when Ca^2+^ was depleted by removing extracellular Ca^2+^ and adding EGTA, we observed an interesting phenomenon. The elevation of [Ca^2+^]_i_ triggered by acidification in the absence of extracellular Ca^2+^ was declined from 2.82 ± 0.20 (n = 25 cells) to 1.03 ± 0.12 (n = 19 cells, *P* < 0.01, Student’s *t*-test, Fig. 5a), whose inhibition ratio was much close to the effects of PcTx1 and CPZ combination (Fig. 1f). This result suggests that ASIC and TRPV1 channels might be the component of extracellular Ca^2+^ entry, and the left elevation of Ca^2+^ might be derived from intracellular Ca^2+^ stores. Since sarcoplasmic reticulum is the possible source of intracellular Ca^2+^ elevation in cardiomyocytes, and thapsigargin (TG) is the inhibitor of SR Ca^2+^-ATPase that can exhaust the Ca^2+^ content, so TG was used here to examine the role of SR. The results showed that the extracellular acidification-induced [Ca^2+^]_i_ elevation in the absence of extracellular Ca^2+^ was almost totally abolished by TG (3 μM) with the inhibition ratio of 90.59 ± 1.46% (n = 15 cells, *P* < 0.01 *vs* control n = 14 cells, Student’s *t*-test, Fig. 5b), indicating that SR mobilization mediates acidosis-induced elevation of Ca^2+^ that derived from intracellular Ca^2+^ stores.

**Figure 6:**
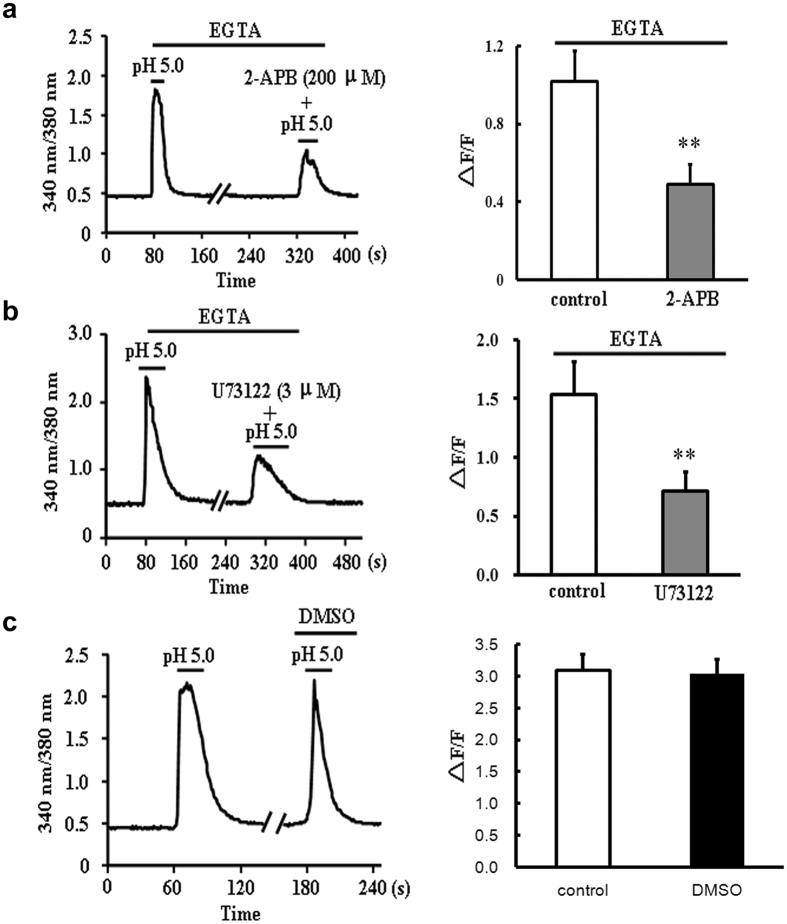
The effects of 2-APB and U73122 on pH 5.0 solution-induced [Ca^2+^]_i_ elevation in the absence of extracellular Ca^2+^. (**a**) Representative 340/380 nm ratio and summary data (∆F/F) of primary cardiomyocytes showing the changes in [Ca^2+^]_i_ induced by pH 5.0 solution in the absence or presence of 2-APB (200 μM) (n = 15 cells for each group). Data were shown as mean ± s.e.m (**P < 0.01 *vs* control, Student’s *t*-test). (**b**) Representative 340/380 nm ratio and summary data (∆F/F) of primary cardiomyocytes showing the changes in [Ca^2+^]_i_ induced by pH 5.0 solution in the absence or presence of U73122 (3 μM) (n = 13 cells for each group). Data were shown as mean ± s.e.m (**P < 0.01 *vs* control, Student’s *t*-test). (**c**) Representative traces of 340/380 nm ratio and summary data (∆F/F) of primary cardiomyocytes showing no changes of [Ca^2+^]_i_ (pH 5.0) in the presence of 0.1% DMSO (conrol: n = 12 cells, DMSO: n = 18 cells, *P* > 0.05 *vs* control, Student’s *t*-test).

We then testify the contribution of SR in the extracellular acidification-triggered [Ca^2+^]_i_ elevation. Since GPCRs are reported to couple to PLC, liberating IP_3_ to bind to IP_3_R, and IP_3_R is a kind of receptor that expressed in SR membrane, mediating Ca^2+^ release^21^, the changes of [Ca^2+^]_i_ with 2-APB, the blocker of IP_3_ receptor (IP_3_R) and U73122, the inhibitor of PLC in cytoplasm were measured here. As shown in Fig. 6a and b, treatment with 2-APB (200 μM) and U73122 (3 μM) significantly decreased the amplitude of Ca^2+^ elevation evoked by extracellular acidification in the absence of extracellular Ca^2+^, the inhibitory ratios were 49.80 ± 4.85% (n = 15 cell) and 53.93 ± 5.14% (n = 13 cells, *P* < 0.01 *vs* control, Student’s *t*-test), respectively. To rule out the influence of DMSO on responses of cardiomyocytes to acidification, we measured the acidic solution (pH 5.0)-triggered Ca^2+^ signals with incubation of 0.1% DMSO, and no significant decay of Ca^2+^ signals was observed after DMSO exposure (n = 18 cells, *P* > 0.05 *vs* control n = 12 cells, Student’s *t*-test, Fig. 6c). Hence, these results suggested that the elevations of intracellular Ca^2+^ triggered by extracellular acidification in the absence of extracellular Ca^2+^ might be due to the activation of PLC/IP3 receptor and the following mobilization of SR.

### The involvement of OGR1 in the extracellular acidification-induced Ca^2+^ mobilization in rat ventricular cardiomyocytes

**Figure 7:**
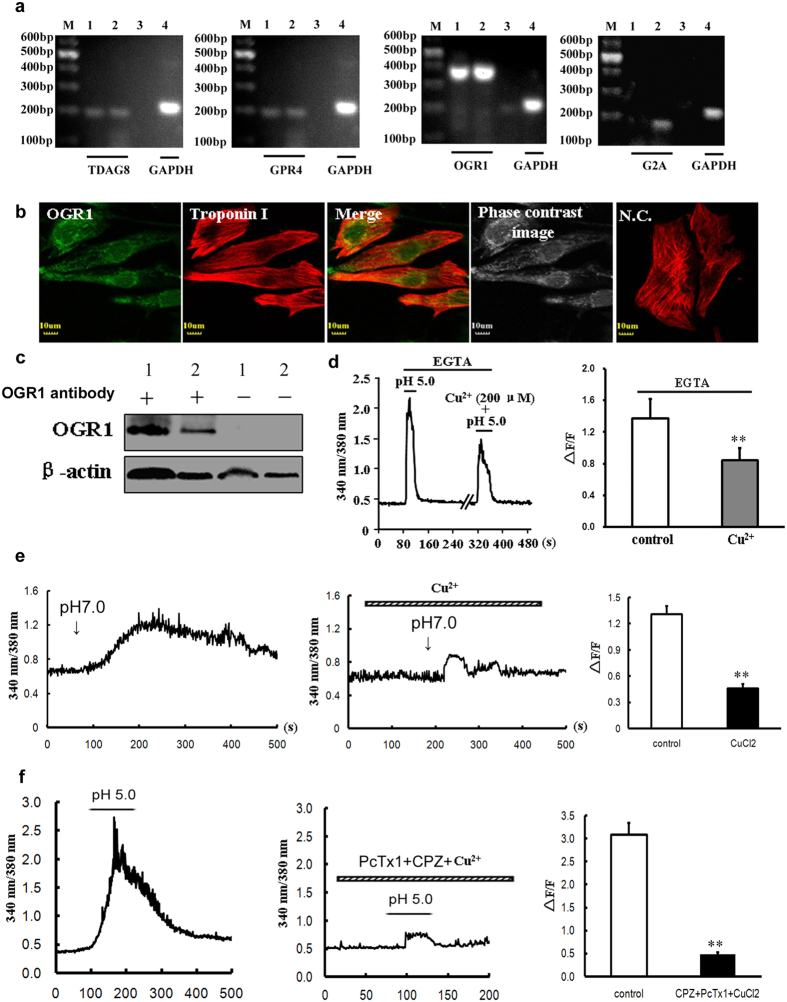
The involvement of OGR1 in the extracellular acidification-induced Ca^2+^ mobilization in rat ventricular cardiomyocytes. (**a**) RT-PCR detection of TDAG8, GPR4, OGR1, and G2A mRNA transcription in cultured rat cardiomyocytes. Spleen tissues were used as positive controls, and samples without the addition of RNA were used as negative controls. M: marker; 1, 4: cardiomyocytes; 2: spleen; 3: negative sample. Representative data were shown from three independent experiments. (**b**) Co-localization of OGR1 (*green*) and Troponin I (*red*) in *in vitro* rat primary cardiomyocytes by double-labeling fluorescence. NC: without primary OGR1 antibody as negative control. Scale bars: 10 μm. Representative images were shown from three independent experiments. (**c**) Western blotting indicating the protein expression of OGR1 in rat cardiomyocytes. Spleen tissues were used as positive controls, and samples without OGR1 antibody were used as negative controls. 1: spleen; 2 cardiomyocytes. Representative blots were shown from four independent experiments. The blots with multiple exposure times were shown in Supplementary Fig. S3. (**d**) Representative 340/380 nm ratio and summary data (∆F/F) of primary cardiomyocytes showing the changes in [Ca^2+^]_i_ induced by pH 5.0 solution in the absence or presence of Cu^2+^ (200 μM). (n = 16 cells for control groups; n = 15 cells for Cu^2+^-treated groups). Data were shown as mean ± s.e.m (**P < 0.01 vs control, Student’s *t*-test). (**e**) Representative [Ca^2+^]_i_ responses and summary data (∆F/F) of primary cardiomyocytes showing the changes of [Ca^2+^]_i_ induced by lowing pH from 7.6 to 7.0 in the absence or presence of Cu^2+^ (100 μM) (n = 15 cells for control groups; n = 9 cells for Cu^2+^-treated groups). Data were shown as mean ± s.e.m (**P < 0.01 vs control, Student’s *t*-test). (**f**) Representative [Ca^2+^]_i_ responses and summary data (∆F/F) of primary cardiomyocytes showing the changes of [Ca^2+^]_i_ induced by pH 5.0 solution in the absence or presence of 20 μM CPZ + 10 nM PcTx1 + 200 μM Cu^2+^ (n = 20 cells for control groups; n = 25 cells for CPZ/PcTx1/Cu^2+^-treated groups). Data were shown as mean ± s.e.m (**P < 0.01 vs control, Student’s *t*-test).

Considering that Gq protein has been accepted as the upstream signal of PLC activation, we asked how extracellular acidification could activate Gq protein. In previous studies, the family of proton sensing GPCRs have been reported to play a role in linking extracellular protons to Gq or Gs signals^19^. Thus, the gene expression of the four subtypes (TDAG8, GPR4, OGR1, and G2A) of GPCRs was examined in primary cultured rat ventricular cardiomyocytes. As shown in Fig. 7a, the specific DNA bands of TDAG8, GPR4, and OGR1 were detected in primary cultured cardiomyocytes, however, G2A product was absence in the same cardiomyocytes. Thus, the gene type of proton sensing G protein coupled receptors was clearly determined in primary cultured ventricular cardiomyocytes.

Among the already confirmed subtypes of G protein coupled receptors (TDAG8, GPR4, and OGR1) in rat ventricular cardiomyocytes, only OGR1 was considered to be the primary protein that transferred extracellular proton signals to Gq proteins^19^. Based on the PCR results, we further investigated the expression of OGR1 in *in vitro* cardiomyocytes using specific OGR1 antibody. As shown in Fig. 7b (also see Supplementary Fig. S3) and 7c, the immunofluorescence and western blotting analyses clearly proved the existence of OGR1 in primary rat ventricular cardiomyocytes. It is reported that OGR1 has the maximal activation at pH 6.8, and pH 7.6 solution will make it more sensitive to pH change^22^, so we changed the extracelluar pH from 7.6 to 7.0 to investigate the OGR1 activation. The results showed a mild elevation of [Ca^2+^]_i_ in cultured myocardiac cells with the peak of ΔF/F 1.31 ± 0.09 (n = 15 cells), and Cu^2+^, the inhibitor of the protonation of extracellular histidines residues in OGR1, could inhibit this elevation to 0.46 ± 0.05 (n = 9 cells, *P* < 0.01 vs control, Student’s *t*-test, Fig. 7e), further indicating the existing of OGR1. Next, to confirm the involvement of OGR1 in the extracellular acidification-triggered [Ca^2+^]_i_ elevation, we evaluated the effects of Cu^2+^ in the absence of extracellular Ca^2+^. Our results showed that Cu^2+^ markedly attenuated the elevation of [Ca^2+^]_i_ triggered by pH 5.0 acidic solutions with the inhibitory ratio of 47.91 ± 8.35% (n = 15 cells, *P* < 0.01 vs control n = 16 cells, Student’s *t*-test, Fig. 7d). Thus, the sensor of extracellular protons in cardiomyocytes that triggered the elevation of [Ca^2+^]_i_ was OGR1. In order to verify both ASIC/TRPV1 channels and OGR1 protein contribute to acidosis-induced Ca^2+^ release, we combined three inhibitors of PcTx1, CPZ and Cu^2+^ together, the results showed nearly complete blockade (84.35% ± 1.36%, n = 25 cells, *P* < 0.01 vs control n = 20 cells, Student’s *t*-test) of Ca^2+^ transient induced by pH 5.0 solution (Fig. 7f). Taken together, there are two components for Ca^2+^ elevation in response to elevated external protons, one is ASIC/TRPV1 channel and another is OGR1protein.

## Discussion

In the present study, we first found that extracellular acidification can induce an elevation of [Ca^2+^]_i_ in cultured rat cardiac myocytes; We then identified that ASICs and TRPV1 were expressed in the cardiac myocytes and jointly mediated extracellular calcium entry; Thirdly, another sensor of extracellular protons OGR1 was also found in cardiomyocytes, it mediated the IP_3_R-gated mobilization of intracellular Ca^2+^ in SR via OGR1-PLC-IP_3_-IP_3_R signaling pathway.

Acidosis is a detrimental condition accompanied with some cardiac disease, for example, myocardial ischemia. The outcome of acidosis is the increase of [Ca^2+^]_i_ followed by various functional changes in cardiomyocytes^1^. In the present study, application of extracellular buffer at pH 6.0 or 5.0 to primary cardiomyocytes also showed a robust elevation of [Ca^2+^]_i_ in a pH-dependent manner. Since H^+^-sensitive ion channels or receptors may be one of the mediators for the influx of extracellular Ca^2+^, the effects of ASICs and TRPV1 were investigated. The antagonists of ASIC and TRPV1 channels inhibited in part the augmentation of [Ca^2+^]_i_, respectively. Meanwhile, blockade of both ASIC and TRPV1 channels inhibited the elevation of [Ca^2+^]_i_ more significantly, and the inhibition ratio was similar to the effect of EGTA, where extracellular Ca^2+^ was absent. Therefore, ASIC and TRPV1 channels contributed prominently to the acid-evoked Ca^2+^ influx.

Although ASICs and TRPV1 are mainly found in the nervous system, both channels have also been reported to express in cardiovascular systems^16^,^17^,^18^,^23^,^24^, however, there is no evidence for their distribution in the heart. Here, we first demonstrated the presence of the mRNAs and proteins of ASIC1, 2 and 3 in the cultured myocardiac cells. Moreover, different from the predominance of ASICs in neuronal membranes, the localization of ASIC subunits in the cardiomyocytes had unique specificity. This variability resulted in disparate ASIC currents in the myocardium. Therefore, we calculated the pH_50_ of ASIC currents in the myocardiocytes, the value of 5.7 was different from any subunits of ASICs in neurons. Since in the central nervous system, the pH_50_ of homomeric ASIC1a channels is 6.2 or 6.8, ASIC1b 5.9, homomeric ASIC2a channels 4.4, while ASIC3 has a biphasic response with a fast desensitizing current followed by a sustained component (pH_50_: 6.7)^9^. As we known, different ASIC subunits form the heterotypic channels, the shape and characteristics including pH_50_ of current would be changed correspondingly^25^. Thus, we presumed that the principal ASIC currents on the heart were produced by heterogenous ASIC channels. Other than ASIC channels, we also identified TRPV1 channel in the cardiomyocytes, which distributed evenly through cytoplasma and nucleus. The decrease of extracellular pH induced TRPV1-like currents in a pH-dependent manner. Similar with cultured neonatal rat cardiomyocytes, both ASIC and TRPV1 - like currents could be induced in acute isolated adult rat cardiomyocytes. The recording of currents in cardiac myocytes by different experimental conditions and developmental phases further confirmed that both cardiac pH-sensitive channels are functional.

In the present study, the depletion of extracellular Ca^2+^ significantly attenuated the increase in [Ca^2+^]_i_ that triggered by extracellular acidification, suggesting the possible contributions of intracellular Ca^2+^ stores in acidosis-induced elevation of [Ca^2+^]_i_. Under absence of extracellular Ca^2+^ conditions, when the Ca^2+^ contents in SR were exhausted by using TG, the elevation of [Ca^2+^]_i_ was almost totally abolished, demonstrating that acidosis elevated [Ca^2+^]_i_ via prompting the release of Ca^2+^ from SR in cardiomyocytes. Although it is well accepted that SR can be mobilized by ryanodine receptor activation^26^, IP_3_ receptor has attracted more and more attentions recently, especially for their functions in cardiomyocytes. The activity of IP_3_ receptors can be directly potentiated by intracellular IP_3_, triggering the release of Ca^2+^ that elicits excitation-contraction coupling or cardiac hypertrophy, and predisposes ventricle to arrhythmia^27^,^28^,^29^,^30^,^31^. In our experiments, blockade of IP_3_ receptor with 2-APB significantly attenuated the acidification-induced [Ca^2+^]_i_ elevation in cardiomyocytes, which was also observed in the specific inhibitor of PLC, U73122-treated groups, thus, IP_3_ receptors should mediate the extracellular acidification-induced [Ca^2+^]_i_ elevation via IP_3_-PLC signaling. However, the possible contributions of RyRs to the [Ca^2+^]_i_ elevation could not be completely ruled out. In fact, there is a clear potential for cross-talking between RyRs and IP3R-mediated Ca^2+^ signaling pathways in heart muscle^32^. Hence, the relative contributions of RyR-gated Ca^2+^ stores to acidification-induced [Ca^2+^]_i_ elevation would be further investigated in cardiomyocytes.

The activation of IP_3_-generated PLC is mediated by Gq protein that are usually coupled with GPCRs^33^,^34^. Here, we asked whether the activation of PLC by extracellular acidification in cardiomyocytes could be resulted from the activation of some Gq-associated GPCRs. Fortunately, a group of proton sensing G protein coupled receptors provided us some important information. The family of proton sensing G protein coupled receptors contains four subtypes: TDAG8, GPR4, OGR1, and G2A. Among them, TDAG8 and GPR4 are mainly Gs-coupled and the latter two subtypes are usually Gq-coupled^19^,^22^. In light of the above characteristics, we investigated the gene expression of these four proteins in primary cultured cardiomyocytes. Interestingly, the level of TDAG8, GPR4 and OGR1 proteins were high, while G2A was absence in primary cultured cardiomyocytes, suggesting that OGR1 was the most potential Gq-associated GPCR for the intracellular Ca^2+^ mobilization from SR by acidification. Using specific antibody for OGR1, we also examined the existence of OGR1 in ventricular cardiomyocytes, and calcium imaging found that OGR1 had a relative high sensitivity to protons, when extracellular pH was lowered from 7.6 to 7.0, it has already been activated. Furthermore, the possible involvement of OGR1 in the proton-induced activation of PLC and the following mobilization of intracellular Ca^2+^ were also tested by application of Cu^2+^. Pre-incubation of Cu^2+^ significantly attenuated the elevation of [Ca^2+^]_i_ via binding to essential histidine residues in OGR1^22^. Although the inhibitory effects of Cu^2+^ was properly non-specific, the specificity of Cu^2+^ for proton sensing GPCRs other than OGR1 has not been reported. Hence, we concluded that OGR1 mediated the IP_3_R-gated mobilization of intracellular Ca^2+^ in SR via sensing extracellular protons.

**Figure 8:**
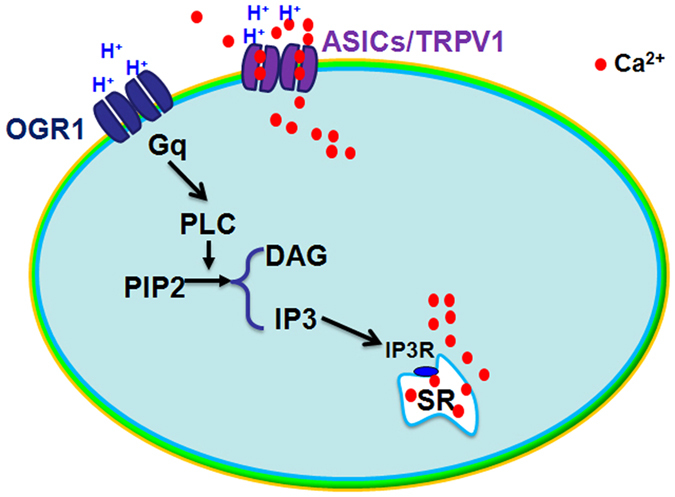
Schematic diagram of mechanisms for the extracellular acidification inducing “Ca^2+^ transients” in cultured rat cardiac myocytes. There are two components for Ca^2+^ elevation in response to elevated external protons, one is ASIC/TRPV1 channel and another is OGR1protein. On one side, ASICs and TRPV1 can be activated by acidosis solution and mediate extracellular Ca^2+^ entry. On the other side, OGR1 mediates mobilization of intracellular Ca^2+^ from SR via OGR1-PLC-IP3-IP3R signaling pathway.

In summary, the present study found that an extracellular acidification can induce“Ca^2+^ transients” in cultured rat cardiac myocytes. As H^+^-sensitive ion channels, ASICs and TRPV1 can be activated by acidosis solution and mediate extracellular Ca^2+^ entry. On the other hand, OGR1 mediates the mobilization of intracellular Ca^2+^ from SR via OGR1-PLC-IP_3_-IP_3_R signaling pathway (Fig. 8). The results and conclusion from the present study will provide a new clue and evidence for understanding the possible roles and mechanisms of H^+^-sensitive receptors in heart ischemia.

## Methods

In these studies, all experimental protocols were conducted in accordance with Guide for the Care and Use of Laboratory Animals published by the National Institutes of Health (NIH Publication, 8^th^ Edition, 2011) and approved by the Committee of Animal Care of Huazhong University of Science and Technology.

### Culture of ventricular myocytes

Firstly, neonatal Sprague-Dawley rats (1–2 days old) were euthanized by decapitation, ventricular tissues were excised and washed three times in phosphate-buffered saline (PBS), and then were cut into shivers with the size less than 1 mm^3^. All shivers were digested at 37 °C in PBS solution containing 0.09% collagenase I (Sigma-Aldrich, St. Louis, MO, USA). Every 3–5 minutes, the digest was sucked into the prepared Dulbecco’s modified Eagle’s medium (DMEM)/F-12 (1:1) (Gibco by Invitrogen, Carlsbad, CA, USA) with 10% fetal bovine serum (Thermo Fisher Hyclone, Logan, UT, USA), and 100 U/ml penicillin – streptomycin to terminate digesting. About repeating 5–6 times, the shivers could be digested completely. The dissociated cells were collected by centrifugation at 118 × g for 6 minutes and suspended again in DMEM/F-12. Bromodeoxyuridine (BrdU, Sigma-Aldrich, St. Louis, MO, USA) at a final concentration of 0.1 mM was added during the first 36 hours to prevent proliferation of cardiac fibroblasts. Myocyte purity was about 95% 48 hours after plating. The cultures were maintained at 37 °C in a humidified 5% CO_2_ atmosphere incubator. Experiments were performed on days of 3–5.

### Acute isolation of rat ventricular myocytes

Rat ventricular myocytes were prepared by enzymatic dissociation^35^. Briefly, male Sprague-Dawley rats (4–6 month) weighing 200–250 g were heparinized and anesthetized with urethane (1 g·kg^−1^ intraperitoneally) before decapitation, and then the heart was removed rapidly and retrogradely perfused with normal Tyrode solution containing (mM): NaCl 135, KCl 5.4, MgCl_2_ 1, CaCl_2_ 1.8, NaH_2_PO_4_ 0.33, HEPES 10, Glucose 10 and pH adjusted to 7.2 with NaOH, and then with nominally Ca^2+^-free Tyrode solution for 5 minutes. Subsequently, Ca^2+^-free Tyrode solution with 0.25 mg/ml collagenase (type I, Sigma-Aldrich, St. Louis, MO, USA), 0.15 mg/ml protease (type E, Sigma-Aldrich, St. Louis, MO, USA) and bovine serum albumin (BSA, Amresco inc, Solon, OH, USA, 1 mg/ml) was perfused through the heart for 8–9 minutes. The perfusate was oxygenated with 100% oxygen and kept constant at 36–37 °C. The digested ventricular myocardium were excised and stored in normal Tyrode solution at room temperature for later experiments.

### Reverse transcription-PCR (RT-PCR) experiment

Total RNA of cultured ventricular myocytes were isolated and cDNA was synthesized with the RevertAid^TM^ FirstStrand cDNA Synthesis system for RT-PCR kits (Fermentas, Burlington, ON, Canada). Methods were similar to our previous report with slightly modified^36^,^37^. Primers used for RT-PCR analysis of all gene expressions were listed on Supplementary Table S1. The cycling parameters were as following: one cycle of 94 °C for 5 minutes and 35 cycles of 94 °C for 15 seconds, 47 °C for 45 seconds, 72 °C for 30 seconds followed by a single 10-minute cycle at 72 °C for extension. RT-PCR products were electrophoresed on a 2% agarose gel by using PCR markers (Tiangen Biotech, China) as the standard to determine the molecular size. Analysis was performed with GENIUS bioimaging system (Kodak, Rochest, NY, USA). Samples without the addition of reverse transcriptase or without the addition of RNA were as negative controls.

### Western - blot experiment

Cultured cardiomyocytes were lysed on ice for 30 min inlyses buffer containing (in mM) 50 Tris - HCl (pH 7.4), 1 EDTA, 100 NaCl, 20 NaF, 3 Na_3_VO_4_, 1 PMSF, and with 1% Nonidet P-40, and protease inhibitor cocktail (Roche, Basel, Switzerland). The lysates were centrifuged at 12000 × g for 15 minutes, and the supernatant was recovered. After denatured, equal amounts of lysate proteins were separated on 10% SDS/PAGE gels, followed by transferred to nitrocellulose membranes (Bio-Rad, Hercules, CA, USA). After blocking, the proteins were probed with the appropriate primary antibodies against ASIC1, ASIC2a, ASIC3, TRPV1 (Alomone labs, Jerusalem, Israel.ASC-014, ASC-012, ASC-018, ACC-030, all in 1:200 dilution) and OGR1 (Santa Cruz Biotechnology, USA. sc-98437). Membrane-bound primary antibodies were detected using proper secondary antibodies conjugated with horseradish peroxidase. Immunoblots were developed on films using the enhanced chemiluminescence technique^37^,^38^ (SuperSignalWest Pico; Pierce Chemical Co., Rockford, IL, USA).

### Immunofluorescence experiment

Ventricular myocytes were fixed with 4% paraformaldehyde in 0.01 M PBS, pH 7.4 for 30 minutes and then rinsed three times with PBS for 10 minutes. Followed by permeabilized with PBS/0.3% TritonX-100 for 30 minutes, cells were blocked with 2% goat serum and 1% BSA in PBS for 1 hour, and then incubated with 1:50 ASIC1, ASIC2a, ASIC3, TRPV1 or OGR1 antibody in PBS/0.3% TritonX - 100/1% BSA/2% goat serum overnight at 4 °C. After rinsed three times in PBS, cells were incubated with 1:100 goat anti-rabbit fluorescein isothiocyanate (FITC)-conjugated (Pierce Chemical Co., Rockford, IL, USA) IgG in PBS containing 0.3% TritonX-100, 2% goat serum and 1% BSA for 1 hour at room temperature. After washed three times in PBS, cells were mounted on glassslides with 50% glycerin and imaged using a confocal laser scanning microscope (FV500; Olympus, Tokyo, Japan). For double-label immunofluorescence, a mouse monoclonal antibody Troponin I (C-4) (Santa Cruz Biotechnology, USA. sc-133117, in 1:50 dilution) was used to mark myocardium with goat anti-mouse Rodamine conjugated (Pierce Chemical Co., Rockford, IL, USA) IgG, or Hoechst33258 was used as nucleus marker.

### Electrophysiological experiments

For cultured rat ventricular myocytes, the beating cells during 3–5 days cultured were selected; for acute isolating cells, quiescent, rod-shape cardiomyocytes showing clear striations were selected to perform patch-clamp recording after 15–20 minutes rest at room temperature. The whole-cell patch-clamp techniques were performed in a voltage-clamp mode with HEKA EPC-10 (HEKA, Munich, Germany) as our previous describe^36^,^38^,^39^. The pipette solution contained (in mM) 140 KCl, 10 NaCl, 1 MgCl_2_·6H_2_O, 5 EGTA, 2 MgATP, 10 HEPES, pH 7.4 with Tris-OH. Cardiac myocytes were voltage-clamped at −80 mV throughout the experiments. A multibarrel perfusion system was used to achieve a rapid exchange of extracellular Tyrode solution (see above), pH adjusted to 7.4, 7.0 and 6.0 with Tris-OH. MES was used instead of HEPES to buffer bath solution pH ranging from 6.0 to 5.0 and 4.0. All experiments were performed at room temperature (22–25 °C).The ASIC current was induced by rapidly lowering pH for 6 seconds and TRPV1 was for 10 seconds.

### Calcium imaging

Cultured cardiac myocytes grown on glass coverslips were washed three times with standard extracellular solutions (150 mM NaCl, 5 mM KCl, 1 mM MgCl_2_·6H_2_O, 2 mM CaCl_2_, 10 mM Glucose, 10 mM HEPES, pH 7.4 with Tris-OH) and incubated with 1 μM Fura-2/AM for 20 minutes at 37 °C. Followed by three washes and additional incubation in standard extracellular solutions for 30 minutes, the coverslips were then transferred to a chamber fixed on the movable stage of an inverted microscope (Olympus IX-70, Tokyo, Japan). Fura-2/AM loaded cells were illuminated at 340 nm for 150 milliseconds and 380 nm for 50 milliseconds at 1-second intervals using a TILL Polychrome monochromator (Munich, Germany). Fura-2 fluorescence emission was imaged at 510 nm by an intensified cooled charge coupled device (TILL Photonics GmbH, Munich, Germany) through an IX-70 fluorescence oil immersion lens (Olympus, Tokyo, Japan) and a 460 nm long-pass barrier filter. Paired F340/F380 fluorescence ratio images were acquired every second for [Ca^2+^]_i_. Ratio images (340/380 nm) were analyzed by TILLVISION 4.0 software. The amplitude of [Ca^2+^]_i_ transient (∆F) represented the difference between baseline concentration (F) and the transient peak response to the stimulation, and [Ca^2+^]_i_ response amplitude was defined as the normalized variations of [Ca^2+^]_i_ (∆F/F)^37^,^39^.

### Statistical analysis

Data are expressed as the mean ± s.e.m. Comparisons were made using Student’s *t* - test with two-tail or ANOVA with LSD. Differences were considered statistically significant at *P* < 0.05 or *P* < 0.01. pH_50_ was fitted by the Hillequation (three parameters): y = a∙ x^b^/(c^b^ + x^b^); a, maximum current density; b, Hill coefficient; c, pH_50_.

## Additional Information

**How to cite this article:** Hu, Y.-L. *et al*. Multiple H^+^ sensors mediate the extracellular acidification-induced [Ca^2+^]_i_ elevation in cultured rat ventricular cardiomyocytes. *Sci. Rep.* **7**, 44951; doi: 10.1038/srep44951 (2017).

**Publisher's note:** Springer Nature remains neutral with regard to jurisdictional claims in published maps and institutional affiliations.

## Supplementary Material

Supplementary Information

## References

[b1] Kapur S., Wasserstrom J. A., Kelly J. E., Kadish A. H. & Aistrup G. L. Acidosis and ischemia increase cellular Ca2+ transient alternans and repolarization alternans susceptibility in the intact rat heart. Am J Physiol Heart Circ Physiol 296, H1491–1512 (2009). 19286955 10.1152/ajpheart.00539.2008

[b2] Mari Y., Katnik C. & Cuevas J. ASIC1a channels are activated by endogenous protons during ischemia and contribute to synergistic potentiation of intracellular Ca(2+) overload during ischemia and acidosis. Cell Calcium 48, 70–82 (2010). 20678793 10.1016/j.ceca.2010.07.002

[b3] Chu L., Greenstein J. L. & Winslow R. L. Modeling Na+ -Ca^2+^ exchange in the heart: Allosteric activation, spatial localization, sparks and excitation-contraction coupling. J Mol Cell Cardiol 99, 174–187 (2016). 27377851 10.1016/j.yjmcc.2016.06.068 PMC5534334

[b4] Salas M. A., Vila-Petroff M. G., Venosa R. A. & Mattiazzi A. Contractile recovery from acidosis in toad ventricle is independent of intracellular pH and relies upon Ca^2+^ influx. J Exp Biol 209, 916–926 (2006). 16481580 10.1242/jeb.02087

[b5] Lascano E. C. . Role of CaMKII in post acidosis arrhythmias: a simulation study using a human myocyte model. J Mol Cell Cardiol 60, 172–183 (2013). 23624090 10.1016/j.yjmcc.2013.04.018

[b6] Said M. . Increased intracellular Ca^2+^ and SR Ca^2+^ load contribute to arrhythmias after acidosis in rat heart. Role of Ca^2+^/calmodulin-dependent protein kinase II. Am J Physiol Heart Circ Physiol 295, H1669–1683 (2008). 18723772 10.1152/ajpheart.00010.2008 PMC2593495

[b7] Krishtal O. The ASICs: signaling molecules? Modulators? Trends Neurosci 26, 477–483 (2003). 12948658 10.1016/S0166-2236(03)00210-8

[b8] Lin S. H., Sun W. H. & Chen C. C. Genetic exploration of the role of acid-sensing ion channels. Neuropharmacology 94, 99–118 (2015). 25582292 10.1016/j.neuropharm.2014.12.011

[b9] Xiong Z. G., Chu X. P. & Simon R. P. Ca2+ -permeable acid-sensing ion channels and ischemic brain injury. J Membr Biol 209, 59–68 (2006). 16685601 10.1007/s00232-005-0840-x

[b10] Yermolaieva O., Leonard A. S., Schnizler M. K., Abboud F. M. & Welsh M. J. Extracellular acidosis increases neuronal cell calcium by activating acid-sensing ion channel 1a. Proc Natl Acad Sci USA 101, 6752–6757 (2004). 15082829 10.1073/pnas.0308636100 PMC404117

[b11] Hoagland E. N., Sherwood T. W., Lee K. G., Walker C. J. & Askwith C. C. Identification of a calcium permeable human acid-sensing ion channel 1 transcript variant. J Biol Chem 285, 41852–41862 (2010). 21036899 10.1074/jbc.M110.171330 PMC3009913

[b12] Benemei S., Patacchini R., Trevisani M. & Geppetti P. TRP channels. Curr Opin Pharmacol 22, 18–23 (2015). 25725213 10.1016/j.coph.2015.02.006

[b13] Mickle A. D., Shepherd A. J. & Mohapatra D. P. Sensory TRP channels: the key transducers of nociception and pain. Prog Mol Biol Transl Sci 131, 73–118 (2015). 25744671 10.1016/bs.pmbts.2015.01.002 PMC5903472

[b14] Nilius B., Owsianik G., Voets T. & Peters J. A. Transient receptor potential cation channels in disease. Physiol Rev 87, 165–217 (2007). 17237345 10.1152/physrev.00021.2006

[b15] Caterina M. J. . The capsaicin receptor: a heat-activated ion channel in the pain pathway. Nature 389, 816–824 (1997). 9349813 10.1038/39807

[b16] Baylie R. L. & Brayden J. E. TRPV channels and vascular function. Acta Physiol (Oxf) 203, 99–116 (2011). 21062421 10.1111/j.1748-1716.2010.02217.x PMC3134601

[b17] Hurt C. M. . Transient Receptor Potential Vanilloid 1 Regulates Mitochondrial Membrane Potential and Myocardial Reperfusion Injury. J Am Heart Assoc 5, 10.1161/JAHA.116.003774 (2016). PMC5079036 27671317

[b18] Yang D.. Activation of TRPV1 by dietary capsaicin improves endothelium-dependent vasorelaxation and prevents hypertension. Cell Metab 12, 130–141 (2010). 20674858 10.1016/j.cmet.2010.05.015 PMC3906919

[b19] Tomura H., Mogi C., Sato K. & Okajima F. Proton-sensing and lysolipid-sensitive G-protein-coupled receptors: a novel type of multi-functional receptors. Cell Signal 17, 1466–1476 (2005). 16014326 10.1016/j.cellsig.2005.06.002

[b20] Russell J. L. . Regulated expression of pH sensing G Protein-coupled receptor-68 identified through chemical biology defines a new drug target for ischemic heart disease. ACS Chem Biol 7, 1077–1083 (2012). 22462679 10.1021/cb300001m PMC3376240

[b21] Hohendanner F., McCulloch A. D., Blatter L. A. & Michailova A. P. Calcium and IP3 dynamics in cardiac myocytes: experimental and computational perspectives and approaches. Front Pharmacol 5, 35 (2014). 24639654 10.3389/fphar.2014.00035 PMC3944219

[b22] Ludwig M. G. . Proton-sensing G-protein-coupled receptors. Nature 425, 93–98 (2003). 12955148 10.1038/nature01905

[b23] Grifoni S. C., Jernigan N. L., Hamilton G. & Drummond H. A. ASIC proteins regulate smooth muscle cell migration. Microvasc Res 75, 202–210 (2008). 17936312 10.1016/j.mvr.2007.08.003 PMC2293954

[b24] Tan Z. Y. . Acid-sensing ion channels contribute to transduction of extracellular acidosis in rat carotid body glomus cells. Circ Res 101, 1009–1019 (2007). 17872465 10.1161/CIRCRESAHA.107.154377

[b25] Wemmie J. A., Price M. P. & Welsh M. J. Acid-sensing ion channels: advances, questions and therapeutic opportunities. Trends Neurosci 29, 578–586 (2006). 16891000 10.1016/j.tins.2006.06.014

[b26] Camors E. & Valdivia H. H. CaMKII regulation of cardiac ryanodine receptors and inositol triphosphate receptors. Front Pharmacol 5, 101 (2014). 24847270 10.3389/fphar.2014.00101 PMC4021131

[b27] Domeier T. L. . IP3 receptor-dependent Ca^2+^ release modulates excitation-contraction coupling in rabbit ventricular myocytes. Am J Physiol Heart Circ Physiol 294, H596–604 (2008). 18055509 10.1152/ajpheart.01155.2007

[b28] Harzheim D. . Increased InsP3Rs in the junctional sarcoplasmic reticulum augment Ca^2+^ τtransients and arrhythmias associated with cardiac hypertrophy. Proc Natl Acad Sci USA 106, 11406–11411 (2009). 19549843 10.1073/pnas.0905485106 PMC2708695

[b29] Harzheim D. . Elevated InsP3R expression underlies enhanced calcium fluxes and spontaneous extra-systolic calcium release events in hypertrophic cardiac myocytes. Channels (Austin) 4, 67–71 (2010). 19934645 10.4161/chan.4.1.10531

[b30] Nakayama H. . The IP3 receptor regulates cardiac hypertrophy in response to select stimuli. Circ Res 107, 659–666 (2010). 20616315 10.1161/CIRCRESAHA.110.220038 PMC2933281

[b31] Proven A. . Inositol 1,4,5-trisphosphate supports the arrhythmogenic action of endothelin-1 on ventricular cardiac myocytes. J Cell Sci 119, 3363–3375 (2006). 16882691 10.1242/jcs.03073

[b32] Fill M. Mechanisms that turn-off intracellular calcium release channels. Front Biosci 8, d46–54 (2003). 12456314 10.2741/924

[b33] Hubbard K. B. & Hepler J. R. Cell signalling diversity of the Gqalpha family of heterotrimeric G proteins. Cell Signal 18, 135–150 (2006). 16182515 10.1016/j.cellsig.2005.08.004

[b34] Sandal M., Paltrinieri D., Carloni P., Musiani F. & Giorgetti A. Structure/function relationships of phospholipases C Beta. Curr Protein Pept Sci 14, 650–657 (2013). 24384033 10.2174/13892037113146660085

[b35] Feng M.. Activation of epidermal growth factor receptor mediates reperfusion arrhythmias in anaesthetized rats. Cardiovasc Res 93, 60–68 (2012). 22028338 10.1093/cvr/cvr281

[b36] Xiong Q. J. . Acid-sensing ion channels contribute to the increase in vesicular release from SH-SY5Y cells stimulated by extracellular protons. Am J Physiol Cell Physiol 303, C376–384 (2012). 22592406 10.1152/ajpcell.00067.2012

[b37] Yu X. W. . Acid-sensing ion channels promote the inflammation and migration of cultured rat microglia. Glia 63, 483–496 (2015). 25377529 10.1002/glia.22766

[b38] Huang C.. Existence and distinction of acid-evoked currents in rat astrocytes. Glia 58, 1415–1424 (2010). 20549751 10.1002/glia.21017

[b39] Hu Z. L.. Disruption of PICK1 attenuates the function of ASICs and PKC regulation of ASICs. Am J Physiol Cell Physiol 299, C1355–1362 (2010). 20826761 10.1152/ajpcell.00569.2009

