# Supplementary material for: Multiple H+ sensors mediate the extracellular acidification-induced [Ca2+]i elevation in cultured rat ventricular cardiomyocytes
Source: Sci Rep. 2017 Mar 23;7:44951. doi: 10.1038/srep44951 (PMC5362981; doi:10.1038/srep44951)
Supplement: Supplementary Information [file srep44951-s1.pdf]

Multiple H^+ sensors mediate the extracellular acidification-induced $[Ca^{2+}]_i$ elevation in cultured rat ventricular cardiomyocytes

Yuan-Lang Hu, Xue Mi, Chao Huang, Hui-Fang Wang, Jian-Ren Song, Qing Shu, Lan Ni, Jian-Guo Chen, Fang Wang and Zhuang-Li Hu.

Supplementary information

Supplementary Table

Table S1. PCR primers for different genes

gene	Forward primer name, sequence (5´-3´)	Reverse primer name, sequence (5´-3´)	PCR products (bp)
ASIC1	TGACATTGGTGGTCAAATGG	ATCATGGCTCCCTTCCTCTT	140 bp
ASIC2	CAACCTTCCAGTATCCAGATCTTC	ACCACTTCATCCACCTTGGTAAC	203 bp
ASIC3	AGGGAGAAGTCCCAAAGCAT	GACACTCCATTCCCAGGAGA	107 bp
TRPV1	CAAGACTCGAGATAGACATGCCA	ACATCTCAATTCCCACACACCTCC	282 bp
TDAG8	ATAGTCAGCGTCCCAGCCAAC	CGCTTCCTTTGCACAAGGTG	200bp
GPR4	CTTCCTCAGCTTCCCAAGTG	CCTGGGCCTCCTTTCTAA AC	200bp
G2A	AAGTGTCCAGAATCCACACAGGGT	AGTAAACCTAGCTTCGCTGGCTGT	161bp
OGR1	GATGGGGAACATCACTGCAGA	AACTGGTGGAAGCGG AAGG	350bp
GAPDH	ACATTGTTGCCATCAACGAC	ACGCCAGTAGACTCCACGAC	210 bp

Supplementary Figures

Figure S1

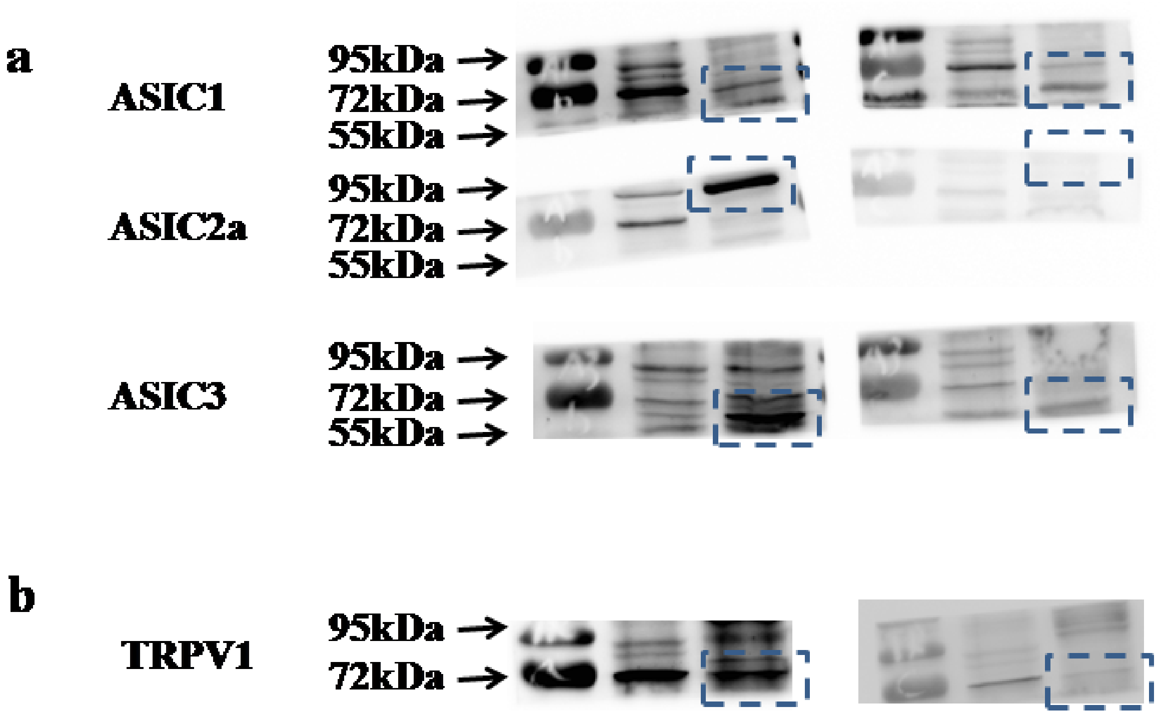

Figure S1. Unedited blots for Fig 2a (a) and Fig 4b (b).

Figure S2

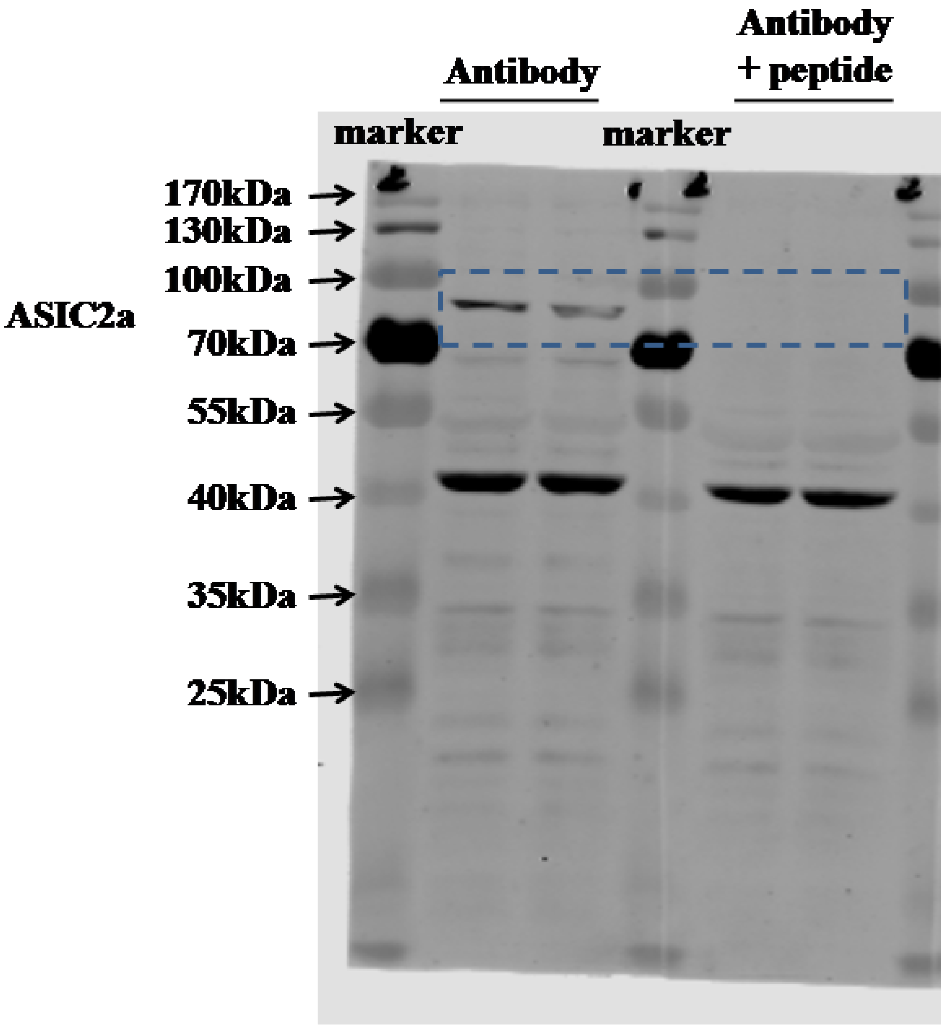

Figure S2. Full-length blots for ASIC2a in cultured cardiac myocytes. Although there were two bands in the full-length gel, only the blot near 70kDa could be blocked by corresponding peptide (right), so the marked bands should be from ASIC2a.

Figure S3

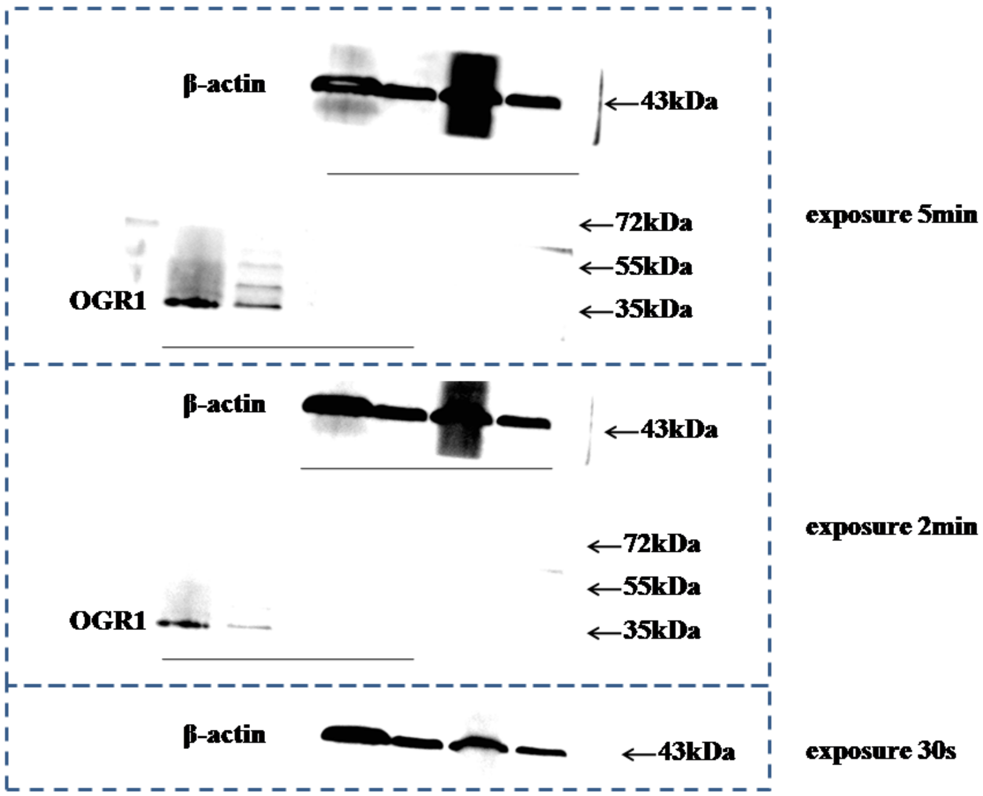

Figure S3. OGR1 blots with multiple exposure times for Fig 7b.
